# Efficacy and safety of novel glycopeptides versus vancomycin for the treatment of gram-positive bacterial infections including methicillin resistant *Staphylococcus aureus*: A systematic review and meta-analysis

**DOI:** 10.1371/journal.pone.0260539

**Published:** 2021-11-29

**Authors:** Wissal Jame, Bilgen Basgut, Abdikarim Abdi

**Affiliations:** 1 Dept. of Pharmacology, University of Zawia, Alzawia, Libya; 2 Dept. of Clinical Pharmacy, Near East University, Lefkosa, Northern Cyprus; 3 Dept. of Pharmacology, Baskent University, Ankara, Turkey; 4 Dept. of Clinical Pharmacy, Yeditepe University, Istanbul, Turkey; Babol University of Medical Science, ISLAMIC REPUBLIC OF IRAN

## Abstract

**Objective:**

To compare between current evidence of novel glycopeptides against vancomycin for the treatment of gram-positive bacterial infections.

**Methodology:**

A systematic review and meta-analysis was done. Major databases were searched for eligible randomized control trials that assessed clinical success, microbiological success and safety profile of novel glycopeptides versus vancomycin for infections caused by gram-positive bacteria.

**Results:**

This meta-analysis included eleven trials (7289 participants) comparing telavancin, dalbavancin and oritavancin with vancomycin. No differences were detected between novel glycopeptides and vancomycin for the treatment of skin and soft tissue infections (SSTIs) among modified intent-to-treat patients (OR: 1.04, CI: 0.92–1.17) as well as within the clinically evaluable patients (OR: 1.09, CI: 0.91–1.30). Data analysed from SSTIs, HAP and bacteremia studies on telavancin showed insignificant high clinical response in microbiologically evaluable patients infected with methicillin resistant *Staphylococcus aureus* (MRSA) (OR: 1.57, CI: 0.94–2.62, *p*: 0.08) and in the eradication of MRSA (OR: 1.39, CI: 0.99–1.96, *P*:0.06). Dalbavancin was non-inferior to vancomycin for the treatment of osteomyelitis in a phase II trial, while it was superior to vancomycin for the treatment of bacteremia in a phase II trial. Data analysed from all trials showed similar rates of all-cause mortality between compared antibiotics groups (OR: 0.67, CI: 0.11–4.03). Telavancin was significantly related with higher adverse events (OR: 1.24, CI: 1.07–1.44, *P*: <0.01) while dalbavancin and oritavancin were associated with significant fewer adverse events (OR: 0.73, CI: 0.57–0.94, *p*: 0.01; OR: 0.72, CI: 0.59–0.89, *p*: <0.01 respectively).

**Conclusion:**

Efficacy and safety profiles of both dalbavancin and oritavancin were the same as vancomycin in the treatment of gram-positive bacterial infections in different clinical settings, while telavancin might be an effective alternative to vancomycin in MRSA infections, but caution is required during its clinical use due to the high risk of adverse events, especially nephrotoxicity.

## Introduction

Staphylococcus aureus (*S*. *aureus)* is a prevailing human pathogen that causes a variety of serious infections ranging from skin and soft tissue infections to life-threatening systemic infection including bloodstream infections [1, 2]. The resistant strain of *S*. *aureus* (MRSA) is still a challenging issue in public health. In the United States (2006), the resistant strain of *S*. *aureus*, MRSA was detected in 60% and 48% of inpatients and outpatients, respectively [3]. Traditionally, MRSA infections have been related to health care-associated risk factors but recently, health care providers have observed that there is also an increasing number of people with community-associated MRSA infections [4].

Recently, vancomycin has been the most frequently prescribed antibiotic in hospitals due to the development of beta-lactams resistant strains of Enterococci and Staphylococci; as a result, it has led to an intense increase in the incidence of completely resistant isolates of *S*. *aureus* to vancomycin that have developed in recent years [5, 6]. A total of 52 Vancomycin Resistant Staphylococcus Aureus (VRSA) strains have been isolated worldwide, since the first strain of VRSA was discovered in Michigan, USA in 2002 [6, 7].

Therefore, to withstand the predicted rise in the morbidity and mortality caused by gram-positive cocci, there has been increased attention on the development of effective and safe alternatives to vancomycin [8]. Continued efforts have led to the development of more potent semisynthetic glycopeptides namely Telavancin, oritavancin, and dalbavancin [8, 9].

In the US and Canada, telavancin was approved in September 2009 for use in the treatment of complicated skin and skin structure infections (CSSSIs) and in June 2013 for use in case of hospital-acquired pneumonia including ventilator-associated pneumonia caused by *S*. *aureus*. In Europe, it was approved on 2 September 2011 as a second-line treatment for hospital-acquired and ventilator-associated pneumonia (VAP), caused by confirmed or suspected MRSA pathogen. However, it was withdrawn in the EU on 23 March 2018 at request of the marketing authorization holder “Theravance”, for commercial reasons [10]. Dalbavancin and oritavancin were approved by the FDA in 2014 for the treatment of SSTIs only; both drugs have long half-lives that allow prolonged dosing intervals [11].

Although there is sufficient data regarding the efficacy and safety of novel glycopeptides for the treatment of different types of infections, most of the current guidelines on gram-positive bacterial infections treatments do not integrate evidence on novel glycopeptides [12, 13].

The aim of this meta-analysis is to systematically evaluate and synthesize the evidence regarding the efficacy and safety of the novel glycopeptide antibiotics versus vancomycin for the treatment of infections caused by gram-positive cocci.

## Methodology

### Study design

This study is a systematic review and meta-analysis of randomized controlled trials (RCTs). It was conducted in accordance with the Preferred Reporting Items for Systematic Reviews and Meta-analyses (PRISMA) checklist and was registered in the Prospero database (CRD42020210331) [14].

### Data source

The following search databases were investigated for pertinent published literature: Medline, Embase, Cochrane Central Register of Controlled Trials (Central) and Clinicaltrials.gov. The search terms applied were ‘‘telavancin OR oritavancin OR dalbavancin” as interventions. To identify unpublished, recently completed research, additional methods were applied which included searching the Inter-science Conference on Antimicrobial Agents, the European Congress of Clinical Microbiology and websites of health technology assessment and related agencies. In addition, relevant articles references were manually searched. The search strategy used to investigate the aforementioned databases did not have any language or time restrictions. The search was conducted in October 2020.

### Search strategy

#### Study selection

Two independent investigators (authors; WJ and AA) carried the literature search, and appropriate studies were investigated for potential inclusion in this meta-analysis. As the gold standard of clinical evidence is randomized controlled trials, only RCTs of any sample size and carried in any year were included in the analysis. Included studies were required to have examined the efficacy or safety of any novel glycopeptide compared with vancomycin for the treatment of patients with any suspected or confirmed bacterial infections caused by gram-positive cocci. Observational studies, animal studies, pharmacokinetic or pharmacodynamics studies and the types of articles that had no primary data including posters, articles reviews, editorials, letters or lacked the interventions of interest were excluded. Finally, eligibility did not require patient hospital admission.

#### Data extraction

The following data were extracted independently (authors; WJ and BB) from each of the studies found: Authors and publication year, study design, number of participants, Intention to treat populations (ITT), modified intention to treat (mITT), clinically evaluable (CE), microbiologically evaluable (ME) with total *S*. *aureus*, Methicillin Sensitive Staphylococcus Aureus (MSSA) and Methicillin Resistant Staphylococcus Aureus (MRSA), infection type, regimen of antibiotics used, period to test of cure (TOC), other clinical, microbiological and safety outcomes.

The intention to treat populations (ITT) included all participants who were randomly assigned to groups, regardless of any deviation from the study protocol. The modified intention to treat populations (mITT) population were those who received at least one dose of the study drug. The clinically evaluable (CE) population was composed of participants who were compatible with all inclusion and exclusion criteria and who provided complete data regarding clinical response outcomes whereas patients who only had gram-negative pathogens were excluded from the population. The microbiologically evaluable (ME) population was the group of patients among the CE population with baseline isolates of gram-positive bacteria. All authors resolved any disagreements by consensus. Table 1 shows the predefined PICOS strategy used for data extraction.

**Table 1:** PICOS strategy for data extraction of novel glycopeptides.

PICOS	Clinical Review
**Participants**	Adult patients (18 years and older) of any gender with any confirmed or suspected bacterial infections caused by gram-positive cocci including MRSA.
**Intervention**	Any novel glycopeptides used in the management of gram-positive bacterial infections will be considered eligible. The antibiotics include: Telavancin, dalbavancin and oritavancin.
**Comparator**	Vancomycin
**Outcome**	Clinical treatment success (resolution of clinical signs and symptoms of infections), microbiological treatment success (eradication of bacteria), adverse events, serious adverse events, discontinuation due to adverse events and mortality.
**Study design**	Randomized controlled trials.

Abbreviation: MRSA: Methicillin resistance *Staphylococcus aureus*.

#### Definitions of infections

Definitions of infections in this meta-analysis were in accordance with the definitions used in the included trials.

- **Skin and soft tissue infections (SSTIs)**: This meta-analysis involved both complicated and uncomplicated SSTIs. It was necessary for one of the following symptoms to exist: swelling, redness, warmth, drainage or discharge, tenderness.
- **Bacteremia**: One of the following signs or symptoms of bacteremia needed to have been present: Temperature ≥ 38.0°C, white blood cell (WBC) count > 10,000 or < 4,000 cells/μL, tachycardia (heart rate > 90 bpm), tachypnea (respiratory rate > 20 breaths/min), hypotension (systolic blood pressure < 90 mmHg).
- **Hospital acquired pneumonia**: Any clinical symptoms (including 2 or more of the following findings: cough, fever, heart rate of ≥120/min, respiratory rate at least 30/min, hypotension <90 mm Hg) acquired after at least 48 hours of patient admission or acquired within 7 days after being discharged from a hospitalization equal to or greater than 3 days were consistent with nosocomial pneumonia.
- **Osteomyelitis**: Osteomyelitis defined by fever, warmth, swelling and redness over the infection area, pain or tenderness upon palpation to bone. Elevated C-reactive protein (CRP), plain radiograph or Magnetic resonance imaging (MRI).

### Quality assessment

The Cochrane tool for assessing risk of bias was used to assess the internal validity of the included RCTs. The tool consists of five domains: randomization, allocation concealment, participants and study personnel blinding, outcome assessors blinding, and selective outcome reporting [15]. A study was considered as high quality if it scored 3 points or more. The quality assessment for unpublished trials could not be done because information on study design was not available from clinical trial registries.

### Analysed outcomes

This meta-analysis included dichotomous outcomes and they were outlined as an odds ratio. The primary outcomes were efficacy-related outcomes that included clinical success (cure, defined as resolution of clinical signs and symptoms of all studied infections in all patients populations) and microbiological success (bacterial eradication in ME with MRSA). Secondary outcomes were safety-related outcomes that comprised overall adverse events (AE) or serious adverse events (SAE), which were probably or possibly related to study treatment and discontinuations due to AEs/SAEs and mortality. Safety related outcomes were measured in mITT populations. Subgrouping analysis was performed according to the type of drug and infection. The clinical success and microbiological success at test of cure (TOC) visits only were included in the meta-analysis.

### Statistical analysis

Data analysis was conducted using the package Meta in R studio (The R Project for Statistical Computing, Version 0.98.501). The total number of patients who experienced the event and the total number of the patients in each arm were directly obtained from the included trials. The random effects model (REM) and fixed effect (FEM) were used to calculate the combined odds ratios (OR) and 95% confidence intervals (CI) by the Mantel-Haenszel method for efficacy and safety outcomes. FEM results were only presented in case of no significant heterogeneity between studies. On the other hand, the REM results were presented [16, 17]. Heterogeneity was measured using both Q statistics and I^2^ tests. A *P* value of Q test <0.05 and I^2^ values of 50–100% were defined as significant heterogeneity. The level of significance of the overall effect size was defined as *p* <0.05. The Egger test was used by the funnel plot method for publication bias assessment, with *P* <0.05 indicating potential bias [18].

## Result

### Studies selection

**Fig 1:**
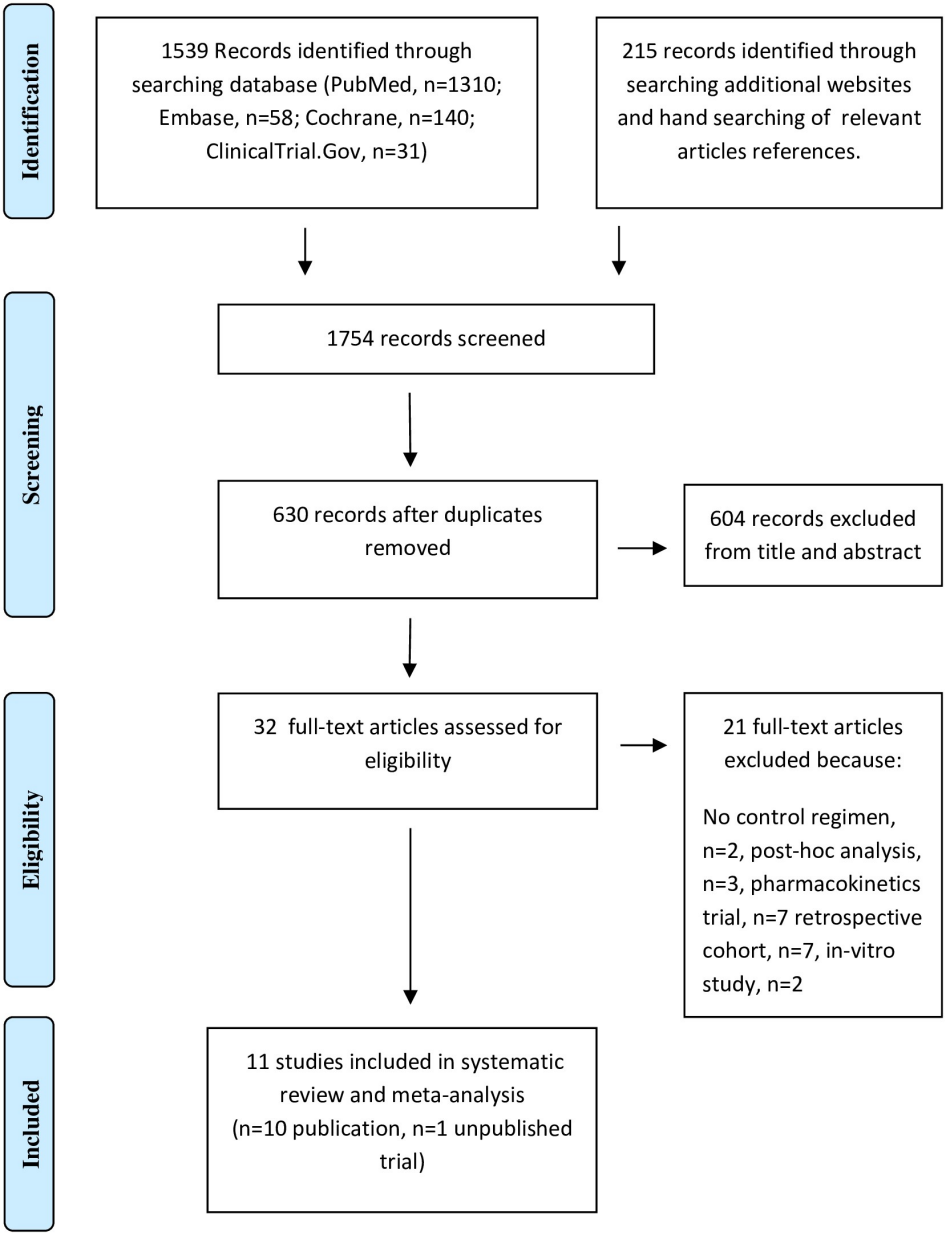
PRISMA flow diagram of the meta-analysis.

The results of the search strategy is shown as a flow diagram in Fig 1. The search of the database produced 1539 records, while the search of additional websites and manual searching of relevant articles references identified 215 further records. Six hundred and thirty potential articles related to this study were identified after removing duplicates. Thirty-two studies were assessed for eligibility and they fulfilled our inclusion criteria according to the information in the title and abstract, of which 21 were excluded due to reasons mentioned in Fig 1. Finally, 11 studies in which 7289 patients were enrolled (3880 were enrolled in telavancin studies while 1959 and 1450 were enrolled in oritavancin and dalbavancin studies, respectively) were included in the meta-analysis: 10 published studies [19–23, 25–29] describing 13 trials comparing novel glycopeptides with vancomycin and one unpublished trial (NCT02208063) [24].

### Studies characteristics

The selected trials and their characteristics are presented in Table 2. All participants had a supposed or confirmed gram-positive infection and the most common type of infection was SSTIs. There were differences among trials regarding study design. Eight studies [19–23, 26, 28, 29] were double-blinded and three were open labels [24, 25, 27]. Furthermore, six studies were phase III [21, 22, 24, 26, 28, 29] and five studies were phase II [19, 20, 23, 25, 27]. Among 11 studies, three included two identical trials [21, 22, 26]. Six studies received quality scores of 5, one study received 4 and the other two scored 3 (Table 2). All RCT assessed patients with SSTIs (5464 patients), HAP (1503 patients), bacteremia (242 patients) or osteomyelitis (80 patients). Regarding telavancin, it was assessed in patients with SSTIs at one dose of 10 mg/kg in two trials [20, 21] and at 7.5 mg/kg once daily in one trial [19]. In addition, telavancin efficacy and safety was assessed in only one study for patients with hospital-acquired pneumonia (HAP) [22]. Regarding bacteremia, two trials evaluated telavancin efficacy and safety at one dose of 7.5 mg/kg and 10 mg/kg in the first and second trial, respectively [23, 24]. Vancomycin was only a comparator in the two trials [21, 22]. However, in three trials, anti-staphylococcal penicillin (oxacillin, cloxacillin, nafcillin) could be used instead of vancomycin if the baseline pathogen was known or suspected to be MSSA [19, 20, 23], daptomycin and synthetic penicillin were other options instead of vancomycin according to the investigators decision in one trial [24]. Two trials only assessed the efficacy and safety of a single 1200 mg dose of oritavancin versus vancomycin in patients with acute bacterial skin and skin structure infection (ABSSSI) [28, 29]. Dalbavancin was studied in four trials; one trial was in bacteremia patients [25], two trials were in SSTIs [26] and one trial was in patients with osteomyelitis [27]. The doses of dalbavancin across those studies were different (The dose for osteomyelitis was higher than for bacteremia and skin infection: 1500mg on day 1 and 1500mg on day 8). Vancomycin was the only comparator in the first trial, oral linezolid was the oral step-down option to vancomycin in the second trial while in osteomyelitis trial, the comparator was the standard care (most commonly IV vancomycin alone or vancomycin IV with switch to linezolid IV or levofloxacin IV to complete 29 days of therapy).

**Table 2:** Characteristics of included trials in meta-analysis.

Study [Ref]	Study design [Quality score]	Infection types	Time to TOC	Sample size	
				Interventions	Comparators
Stryjewski 2005 [19]	R/MC/Ph2/DB [5]	CSSSIs	7–14	TELA(n = 84)	VAN(n = 63)
Stryjewski 2006 [20]	R/MC/Ph2/DB [5]	CSSSIs	7–14	TELA(n = 100)	VAN(n = 88)
Stryjewski 2008 [21]	2R/MC/Ph3/DB [4]	CSSSIs	7–14	TELA(n = 928)	VAN(n = 939)
Rubinstein 2011 [22]	2R/MC/Ph3/DB [5]	HAP	7–14	TELA(n = 749)	VAN(n = 754)
Stryjewski 2014 [23]	R/MC/Ph2/DB [5]	Bacteremia	84	TELA(n = 29)	VAN(n = 29)
NCT02208063 [24]	R/MC/Ph3/OL [–]	Bacteremia	60	TELA(n = 57)	VAN(n = 60)
Raad I 2005 [25]	R/MC/Ph2/OL [3]	Bacteremia	21	DAL(n = 33)	VAN(n = 34)
Boucher 2014 [26]	2R/MC/Ph3/DB [5]	ABSSIs	7–14	DAL(n = 652)	VAN(n = 651)
Rappo U 2019 [27]	R/SC/Ph2/OL [3]	Osteomyelitis	42	DAL(n = 70)	VAN(n = 10)
Corey 2014 [28]	R/MC/Ph3/DB [5]	ABSSIs	7–14	ORITA(n = 475)	VAN(n = 479)
Corey 2015 [29]	R/MC/Ph3/DB [5]	ABSSIs	7–14	ORITA(n = 503)	VAN(n = 502)

Abbreviations: ABSSIs: acute bacterial skin and skin structure infection, CSSSI: complicated skin and skin structure infection, DB: double blind, Dal: dalbavancin, HAP: hospital acquired pneumonia, MC: multicenter, OL: open label, Orita: oritavancin, Ph: phase, Ref: references, R: randomized, SC: single centre, TEL: telavancin, Van: vancomycin.

The vancomycin dose was 1g IV twice daily in all trials. Patients with mixed infections in all trials received a proper regimen of appropriate antibiotics (e.g., aztreonam or other beta-lactams). Patients in all trials, who received systemic anti-staphylococcal antibiotics for more than 48 hours within 7 days before randomization, were excluded from the analysis, except for those who documented resistance to the prior systemic antibiotics.

### Assessment of efficacy and safety of telavancin

There were no differences between telavancin and vancomycin when data regarding the mITT population was analysed (OR: 1.02, CI: 0.89–1.17). In addition, its subsets of patients with SSTIs (OR: 1.08, CI: 0.89–1.32), pneumonia (OR: 0.97, CI: 0.79–1.19) and bacteremia (OR: 0.62, CI: 0.21–1.89) showed the same effect. Telavancin and vancomycin were comparable in the CE population (OR: 1.07, CI: 0.85–1.34) as well as in its subset of patients with skin and soft tissue infections (OR: 1.10, CI: 0.82–1.48), patients with pneumonia (OR: 1.12, CI: 0.75–1.66) and patients with bacteremia (OR: 0.76, CI: 0.35–1.63). Telavancin and vancomycin were associated with the same response in the ME population with total *S*.*aureus* (OR: 1.18, CI: 0.92–1.53). However telavancin was related with numerically higher treatment success in the ME population infected with MRSA (OR: 1.57, CI: 0.94–2.62, *p*: 0.08). The eradication rate of MRSA was insignificantly higher in the telavancin group (OR: 1.39, CI: 0.99–1.96, *P*: 0.06) while it was comparable with vancomycin in the eradication of MSSA (OR: 1.21, CI: 0.70–2.11). There was no difference regarding mortality between the telavancin and vancomycin groups (OR: 1.07, CI: 0.84–1.36). Telavancin was significantly related with higher adverse events (OR: 1.24, CI: 1.07–1.44; *P*: <0.01), serious adverse events (OR: 1.36, CI: 1.14–1.63; *P*: <0.01) as well as with higher discontinuation due to adverse events (OR: 1.47, CI: 1.13–1.91; *P*: <0.01). More episodes of nausea (OR: 1.82, CI: 1.50–2.21; *p*: <0.01) and vomiting (OR: 1.90, CI: 1.43–2.53; *p*: <0.01) were reported with telavancin treatment. Also telavancin was significantly associated with more episodes of hypokalaemia (OR: 1.68, CI: 1.18–2.38; *p*: <0.01) and elevated serum creatinine level (OR: 2.10, CI: 1.62–2.73; *p* = <0.01).

### Assessment of efficacy and safety of dalbavancin

Clinical response was comparable between dalbavancin and vancomycin in only one trial [25] that provided relevant data on mITT patients (OR: 4.64, CI: 0.37–57.93). The same effect was true for CE patients (OR: 3.25, CI: 0.51–20.95) as well as in the subgroup of patients with skin and soft tissue infections (OR: 1.00, CI: 0.56–1.77), osteomyelitis (OR: 4.64, CI: 0.37–57.93), while it showed significant efficacy in bacteremia patients (OR: 15.89, CI: 1.73–145.79; *P*: 0.01). Dalbavancin was associated with almost the same treatment success in the ME population with all *S*.*aureus* and MRSA (For *S*.*aureus*, OR: 0.89, CI: 0.41–1.93, for MRSA, OR: 0.40, CI: 0.08–2.00). Moreover, dalbavancin had a similar efficacy as vancomycin in early clinical response within 48-72h in mITT patients with SSTIs. Only two deaths were reported in the patients receiving dalbavancin compared with seven in patients receiving vancomycin (OR: 0.18, CI: 0.03–0.98). Dalbavancin and vancomycin were related with the same rate of serious adverse events (OR: 0.50, CI: 0.13–1.87) and discontinuation due to adverse events (OR: 0.75, CI: 0.38–1.50). However, dalbavancin was related with significantly less adverse events than vancomycin (OR: 0.73, CI: 0.57–0.94; *p*: 0.01).

### Assessment of efficacy and safety of oritavancin

Only two trials compared oritavancin with vancomycin for the treatment of patients with SSTIs [28, 29]. Among the compared antibiotics, no difference was found among patient populations (mITT patients at TOC, OR: 1.08, CI: 0.86–1.35, mITT patients at early clinical response (48-72h), OR: 1.02, CI: 0.69–1.51, CE patients, OR: 1.01, CI: 0.71–1.45). Mortality rate (OR: 0.67, CI: 0.11–4.03), serious adverse events (OR: 0.96, CI: 0.55–1.68) and treatment related withdrawals (OR: 0.87, CI: 0.58–1.31) were similar between compared antibiotics groups. However, oritavancin was related with significantly less adverse events than vancomycin (OR: 0.72, CI: 0.59–0.89; *p*: <0.01).

### Overall assessment

#### Efficacy

**Fig 2:**
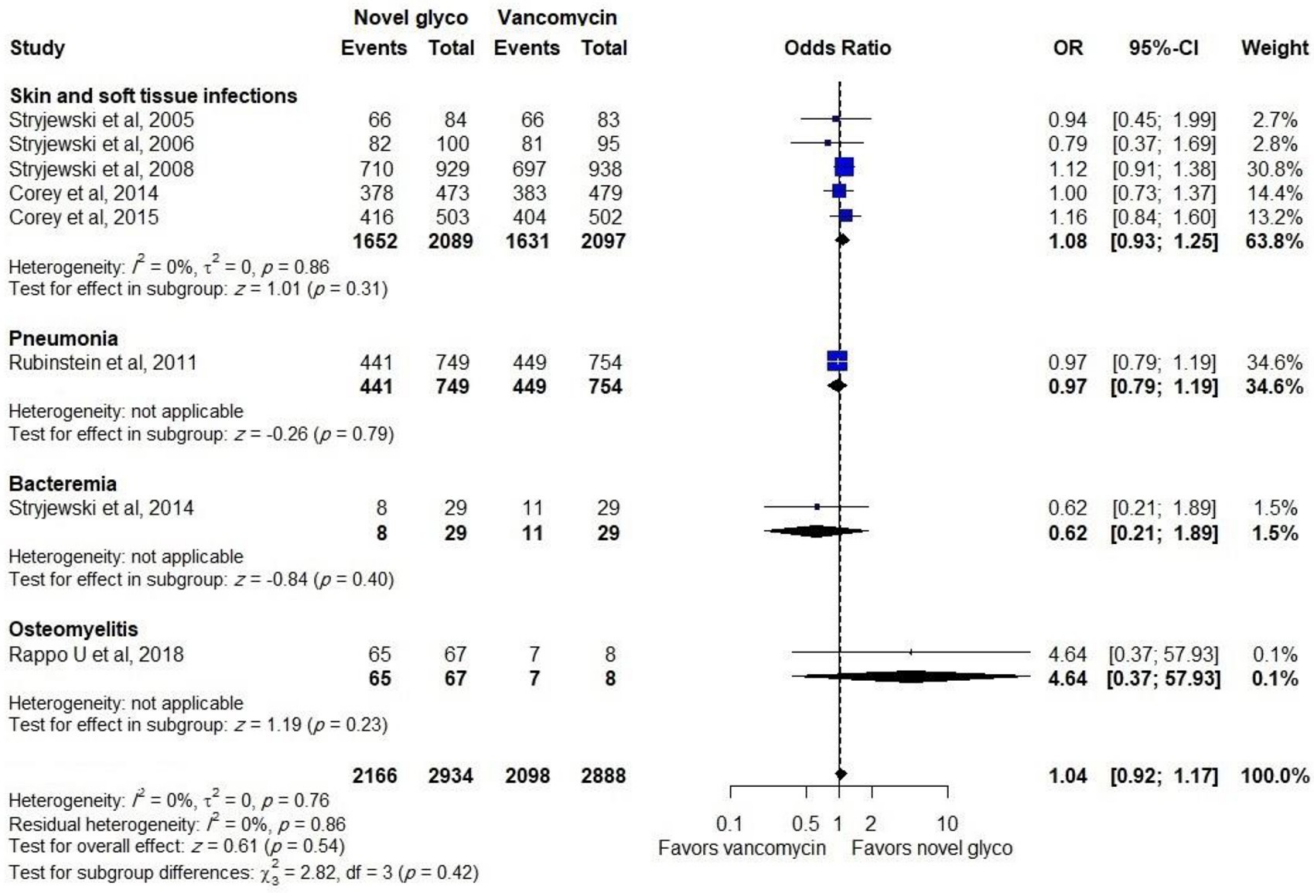
Clinical response of novel glycopeptides against vancomycin in the modified intent-to-treat populations.

Out of 11 trials, data regarding the effectiveness of compared antibiotics in the mITT population were provided in 8 trials, where no difference was found between the novel glycopeptides and vancomycin groups (OR: 1.04, CI: 0.92–1.17). Furthermore, comparable efficacy was reported in the treatment of SSTIs (OR: 1.08, CI: 0.93–1.24), as shown in Fig 2.

**Fig 3:**
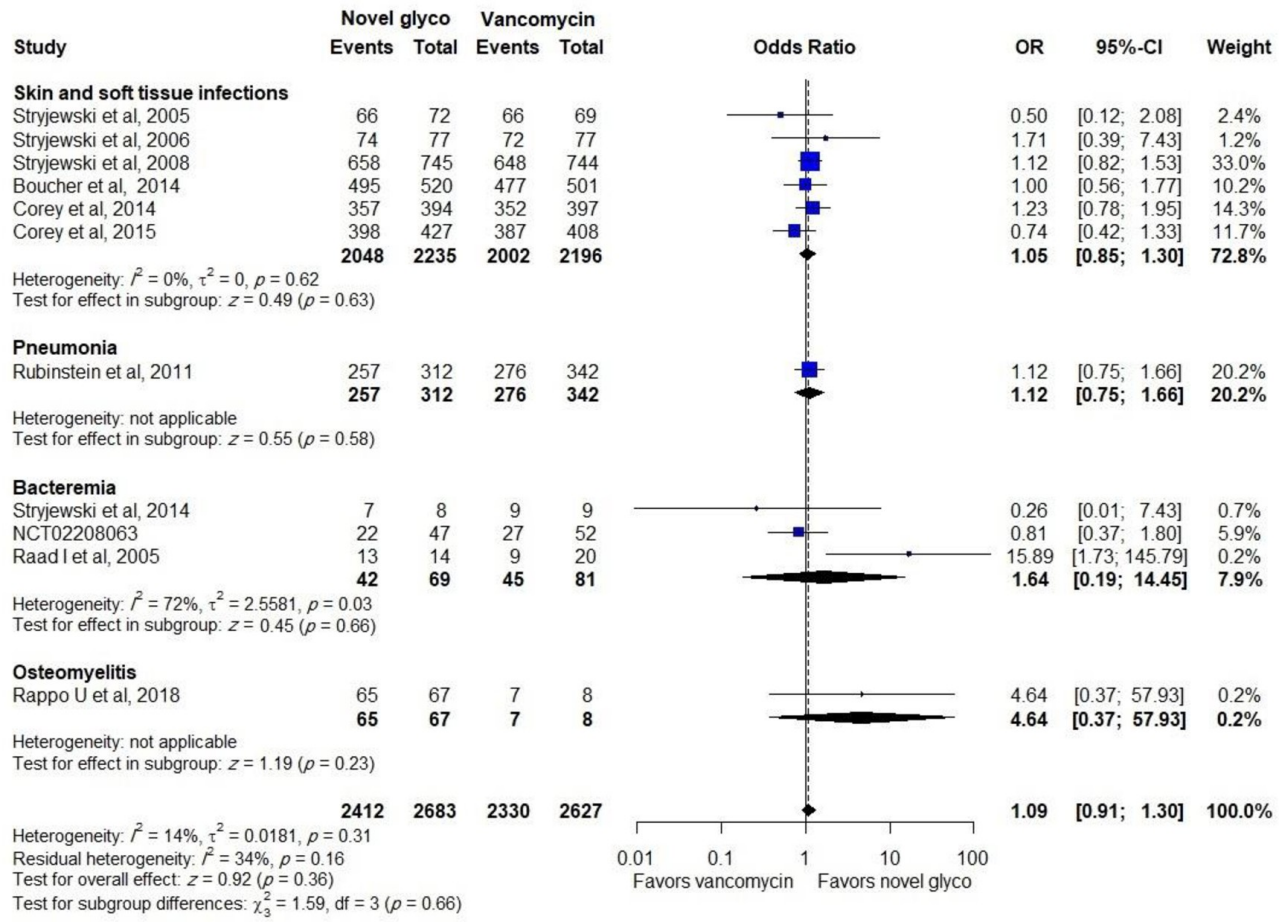
Clinical response of novel glycopeptides against vancomycin in the clinical evaluable populations.

Novel glycopeptides provided the same clinical response rate as vancomycin when data on effectiveness in the CE population from all trials were included in the analysis (OR: 1.09, CI: 0.91–1.30). In addition, a similar effect was observed in the subgroup of patients with SSTIs (OR: 1.05, CI: 0.85–1.30) and bacteremia (OR: 1.27, CI: 0.66–2.46), as shown in Fig 3.

**Fig 4:**
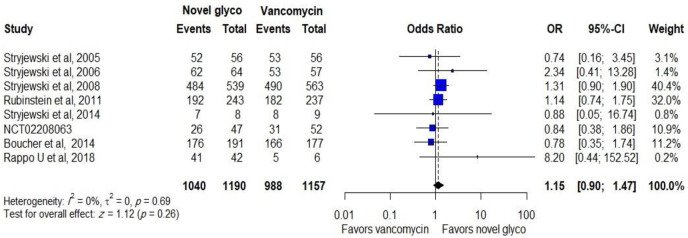
Clinical response of novel glycopeptides against vancomycin in the microbiological evaluable populations with MRSA.

Data regarding treatment success in ME patients with a total number of *S*. *aureus* at the TOC was reported in 8 trials with 2,347 participants (6 trials studied the efficacy of telavancin and 2 trials studied the efficacy of dalbavancin), novel glycopeptides provide same treatment success with (OR: 1.15, CI: 0.90–1.47), as shown in Fig 4. Similar effectiveness was observed between comparators in ME patients with MRSA (OR: 1.36, CI: 0.84–2.18).

#### Safety

**Fig 5:**
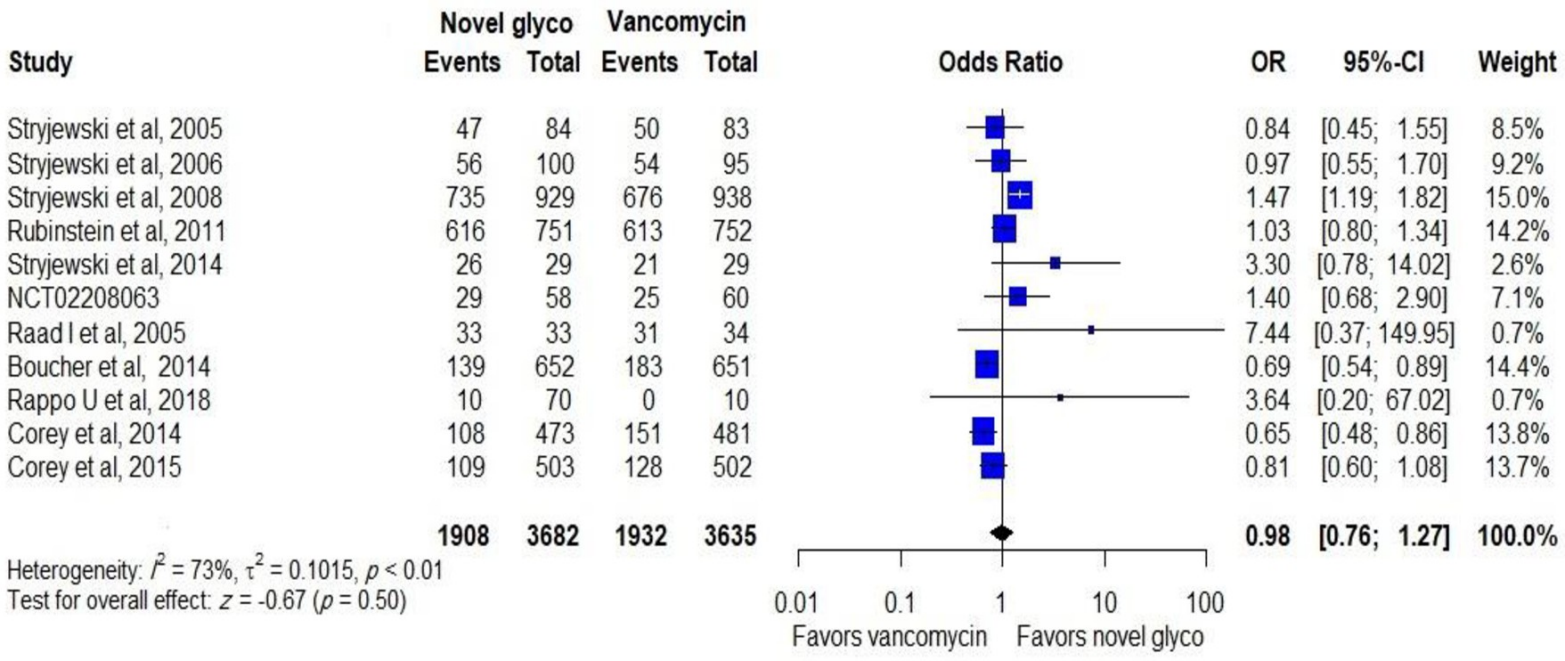
Adverse events of novel glycopeptides against vancomycin in the modified intent-to-treat populations.

The safety was assessed by the incidence of mortality, adverse events, serious adverse events and discontinuance of the treatment due to adverse events. Novel glycopeptides antibiotics were related with fewer adverse events than vancomycin (OR: 0.98, CI: 0.76–1.27) when data from all trials were analysed, as shown in Fig 5.

**Fig 6:**
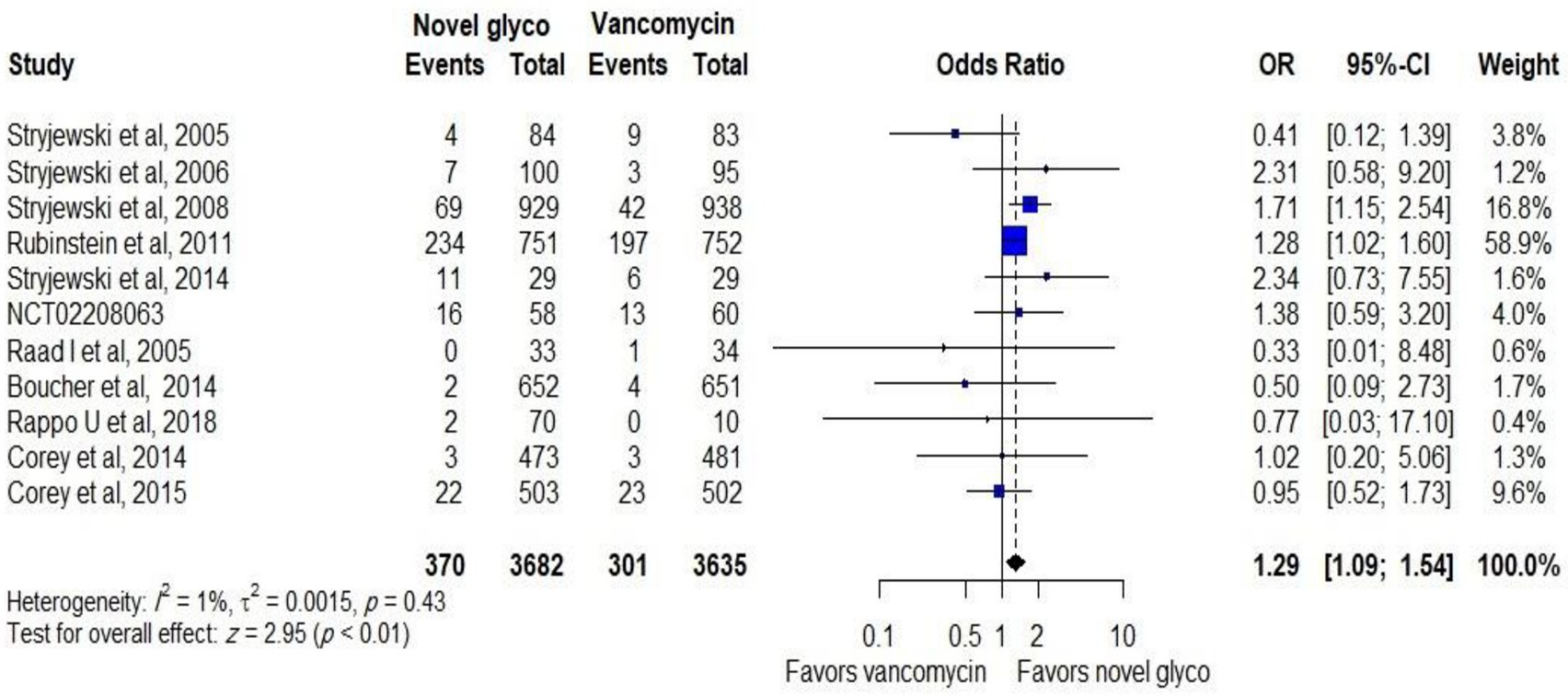
Serious adverse events of novel glycopeptides against vancomycin in the modified intent-to-treat populations.

Serious adverse events were assessed in both groups, where 7317 patients were included in the analysis in 11 RCTs. The pooled analysis showed that serious adverse events were significantly higher in novel glycopeptides users than vancomycin users (OR: 1.29, CI: 1.09–1.54; *p*: <0.01), as shown in Fig 6.

**Fig 7:**
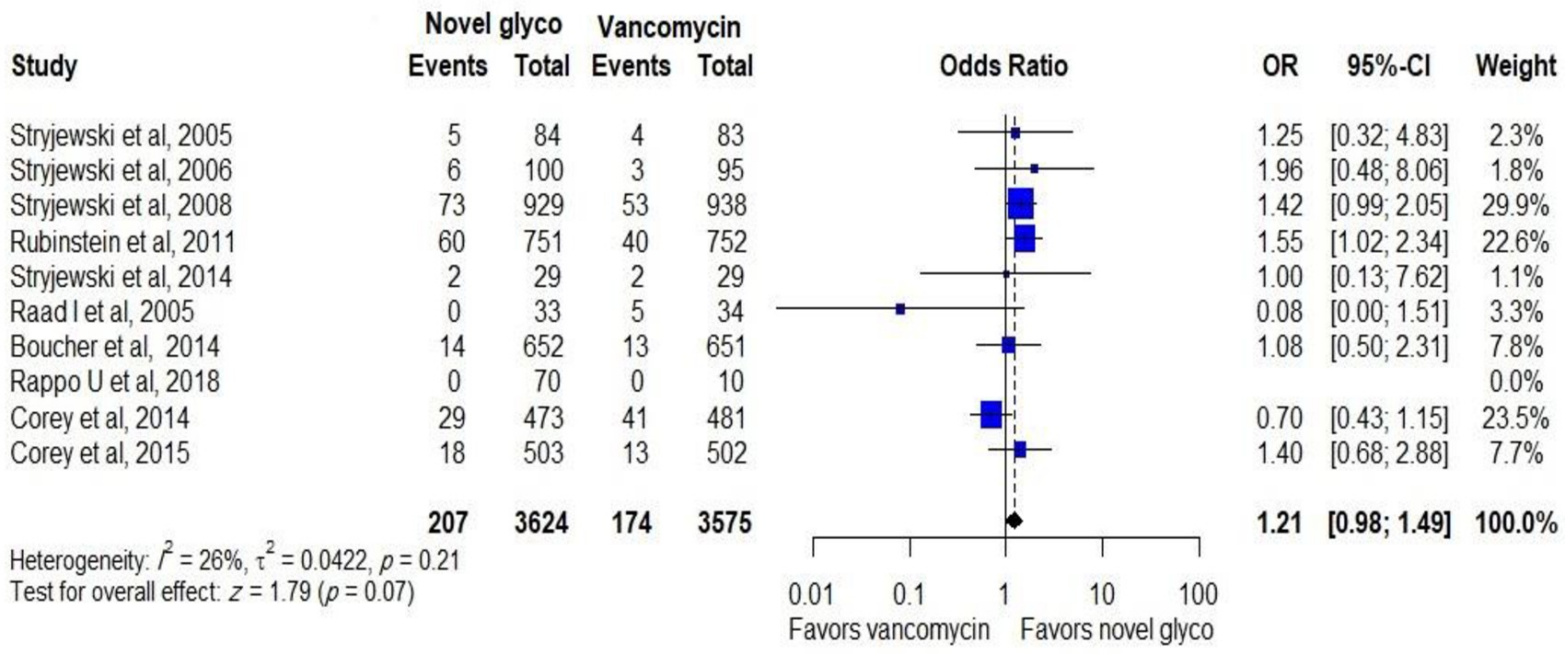
Discontinuation of novel glycopeptides against vancomycin due to adverse events in the modified intent-to-treat population.

A total of 207 out of 3554 patients discontinued novel glycopeptides while 174 patients out of 3565 patients discontinued vancomycin. The pooled analysis showed that the compared antibiotics were related with comparable discontinuation of treatments (OR: 1.21, CI: 0.98–1.49), as shown in Fig 7.

**Fig 8:**
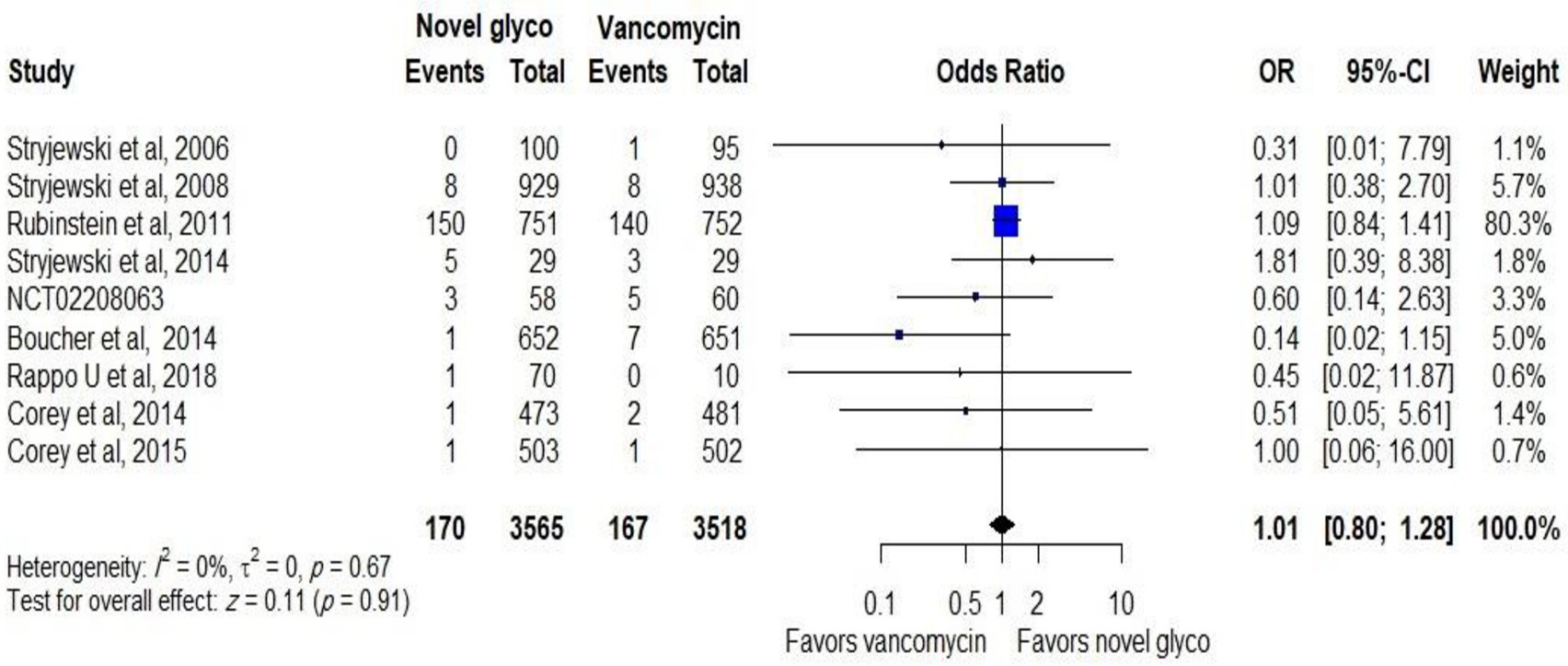
Mortality rate related to novel glycopeptides against vancomycin in the modified intent-to-treat population.

No difference was reported between novel glycopeptides and vancomycin when all trials that provide data on mortality were analysed (OR: 1.01, CI: 0.80–1.28), as shown in Fig 8.

Specific adverse events caused by compared antibiotics are summarized in Table 3. There was no significant difference in the proportions of patients who developed adverse events in the GIT, liver function and hematologic system. The incidence of hypokalaemia and increased serum creatinine level was significantly higher in patients receiving telavancin than in the vancomycin groups. The pruritus incidence was significantly associated with vancomycin compared to novel glycopeptides (OR: 0.52, CI: 0.39–0.69, *P*: <0.01).

**Table 3:** Summary of specific adverse events compared between novel glycopeptides and vancomycin.

Adverse events	No of trials	OR and 95% CI	P-value	Heterogeneity	
				I^2^	P-value
**Headache**	7	1.11 (0.91–1.35)	0.32	0	0.94
**Constipation**	8	1.14 (0.93–1.39)	0.21	35	0.15
**Nausea**	10	1.21 (0.86–1.71)	0.28	67	<0.01
**Vomiting**	7	1.17 (0.70–1.95)	0.53	62	0.02
**Diarrhea**	9	0.89 (0.74–1.09)	0.26	15	0.31
**Pruritus**	6	0.52 (0.39–0.69	**<0.01**	34	0.18
**Anemia**	6	0.81 (0.61–1.07)	0.15	0	0.84
**Hypokalemia**	6	1.79 (1.26–2.53)	**<0.01**	24	0.25
**Leukopenia**	4	0.62 (0.30–1.29)	0.21	0	0.45
**Liver function test**	8	0.84 (0.52–1.35)	0.48	66	<0.01
**Serum creatinine**	6	2.10 (1.61–2.73)	**<0.01**	0	0.45

### Risk of publication bias assessment

**Fig 9:**
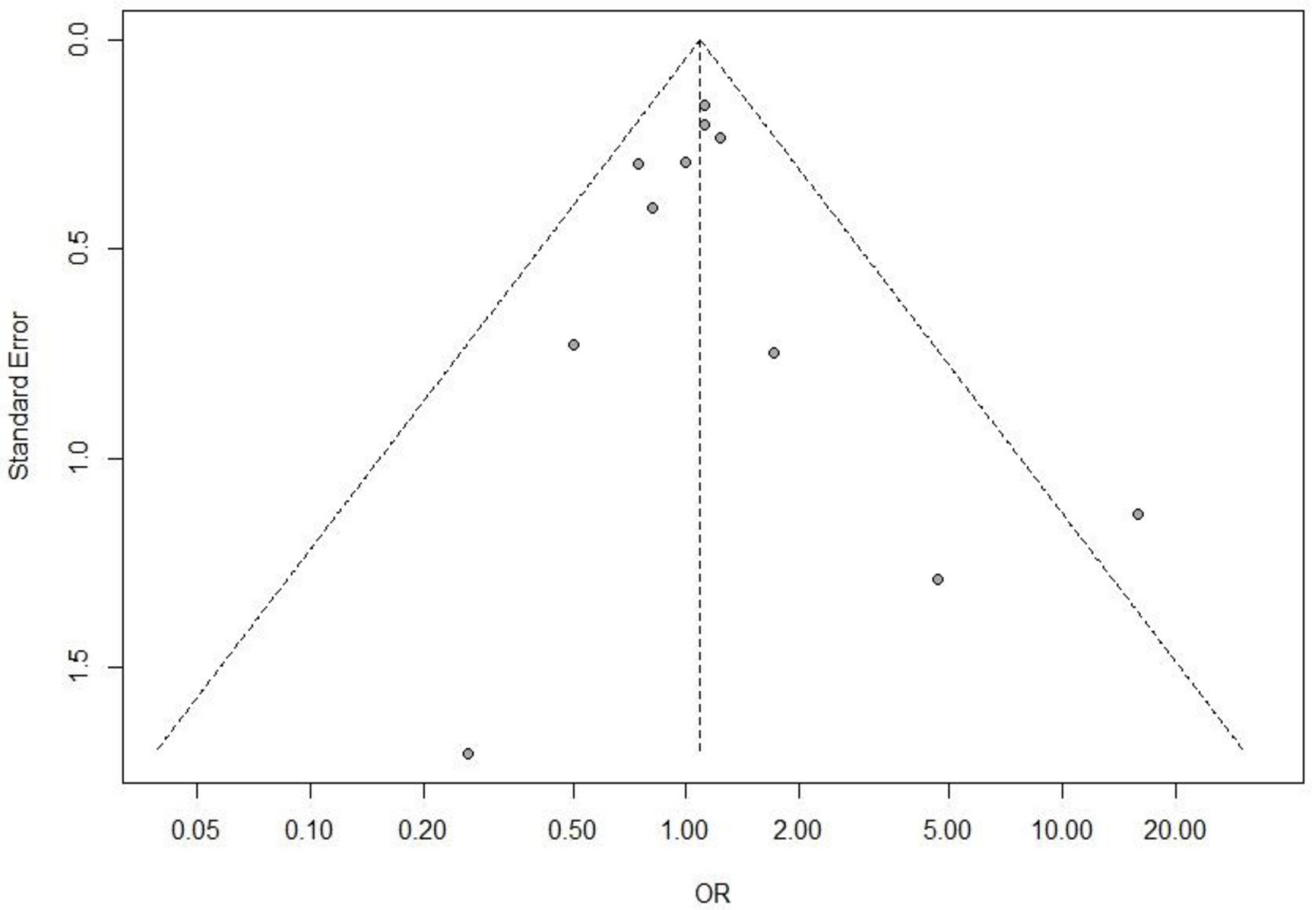
Funnel plot, using data from 11 trials of novel glycopeptides therapy in patients with gram positive cocci.

The applied linear regression relationship between the effect estimates (OR) and the standard error (SE) for funnel plot asymmetry did not indicate any publication bias in the study selection, as shown in Fig 9. *p* values of the Egger test were 0.6225, 0.881, 0.478, and 0.08522 for treatment success in CE, mITT populations, adverse events and mortality respectively.

## Discussion

Our meta-analysis suggests that novel glycopeptides antibiotics are as effective as vancomycin for the treatment of patients with SSTIs, pneumonia, bacteremia and osteomyelitis caused by presumed or confirmed gram-positive bacteria. The analysis of all patient populations (mITT, CE and ME populations) showed no differences between compared antibiotics. In the analysis of ME patients with MRSA, Telavancin was numerically superior to vancomycin (P: 0.08). Moreover, telavancin showed insignificant higher efficacy (*p*: 0.06) when data regarding MRSA eradication was analysed. Although 14% of participants in the studies that evaluated efficacy of telavancin had a trough vancomycin level of ≤ 5μg/mL, which is less than the trough level recommended by (IDSA) [13], there was no obvious evidence showing a relationship between higher effectiveness of telavancin and development of resistance due to under-dosed vancomycin. Moreover, there is insufficient data from several trials supporting the assumption that the lack of monitoring of vancomycin serum levels may have an effect on the outcomes in favor of telavancin. These findings were supported by prior meta-analysis studies that compared telavancin with vancomycin among patients with SSTI and hospital acquired pneumonia [30–33].

Our results demonstrate that dalbavancin and oritavancin were related with fewer adverse events than vancomycin while adverse events and withdrawal due to adverse events were significantly higher in telavancin users than vancomycin users. The most prevalent serious adverse events in the telavancin-receiving patients were elevated serum creatinine and hypokalaemia. All included trials that assessed telavancin safety demonstrated that elevated serum creatinine was more prevalent in patients with impaired renal function at baseline or concomitant use of medications known to affect kidney function. However, in almost all patients who developed renal impairment, the serum creatinine level returned to normal values during the follow-up period (usually within 14 days after discontinuing the study medication). Thus, monitoring of creatinine serum level should be ensured, and a benefit-risk approach should be adopted before giving telavancin to patients at high risk of renal AE. Discontinuation of telavancin therapy was common in patients who had nausea and vomiting. In the light of this, monitoring the safety of telavancin seems warranted.

Of 11 trials included in the meta-analysis, only 6 reported outcomes for SSTIs, where 3 of these trials (2229 mITT, 1784 CE patients) provided evidence about the effectiveness of telavancin against vancomycin. The pooled odds ratio showed similar clinical response rates between telavancin and vancomycin for the treatment of patients with SSTIs. In addition, Stryjewski et al. [34] conducted post hoc analysis of ATLAS trials and documented that telavancin provided the same clinical response as vancomycin in patients with CSSTIs including infections caused by MRSA. A retrospective analysis of the ATLAS study conducted by Wilson SE et al. reported that clinical cure and microbiological eradication rates exhibited consistent trends favouring telavancin over vancomycin; however, the differences were not statistically significant [35].

The efficacy of oritavancin for treating ABSSSI was assessed in two trials. 1626 CE patients and 1959 mITT patients were evaluated at TOC and 48-72h respectively. A single 1200-mg dose of oritavancin showed similar efficacy as 7–10 days of twice-daily vancomycin; this result was consistent with a meta-analysis performed by Thom H et al. [36]. Although one dose of oritavancin with its extended elimination half-life was not associated with any serious adverse events during follow-up assessment [37], the FDA issued a warning [38] about the development of more cases of osteomyelitis in the oritavancin treated arm than in the vancomycin-treated arm. In comparison with telavancin and dalbavancin, oritavancin is the only lipoglycopeptide that has potent activity against VRSA because it is capable of working through multiple modes of action [39, 40].

One study (including two identical trials) [26] tested two doses of dalbavancin against vancomycin for the treatment of ABSSSI. There was no difference between dalbavancin and vancomycin when 1010 CE patients were assessed at TOC. In addition, the evaluation of 1312 mITT patients at 48-72h showed similar clinical responses. The results of prior network meta-analyses [32, 41] support our findings. Some concerns have been raised about the development of potential resistance during the course of treatment due to extended exposure to sub-therapeutic levels, and our assumption was supported by two recent studies [42, 43], which found that dalbavancin non-susceptible VRSA strain resistance was developed in patients with an MRSA. Two doses of dalbavancin were tolerated and as effective as linezolid given twice daily for 14 days for the treatment of patients with complicated ABSSSI, including those infected with MRSA [44]. In addition, a phase III randomized double-blind clinical trial of dalbavancin conducted by Dunne MW et al. [45] was conducted with 698 patients. In this study, a single 1500 mg infusion of dalbavancin was found to be non-inferior to the 2-dose regimen of 1000 mg IV on day 1 and 500 mg IV on day 8, which led to FDA and EU approval of the single 1500 mg dose for ABSSSI, in addition to the 2-dose regimen.

A total of 1503 mITT and 654 CE patients were analysed in one study comparing telavancin with vancomycin for patients with hospital-acquired pneumonia, revealing that telavancin has a similar cure rate to vancomycin. Although the ATTAIN trials were not optimally designed for a mortality end point, the increased mortality rates was an important issue in understanding telavancin efficacy for the treatment of HAP. However, mortality rates were higher in patients with prior moderate to severe renal impairment (CrCl, ≤30 mL/min) and in patients with mixed infections who did not receive adequate gram-negative therapy. This observation was supported by post hoc analysis conducted by Lacy and colleagues [46]. Vancomycin and linezolid were the only two antibiotics that had been approved for the treatment of hospital-acquired pneumonia (HAP) and ventilator-associated pneumonia (VAP) caused by MRSA as of 2013 [47]. Therefore, the use of telavancin should be considered in cases where first line agents are not suitable. Furthermore, 34% of participants in the vancomycin group had trough vancomycin levels of 10 μg/mL, which is less than the trough level recommended by IDSA. In this regard, it was not possible to say that telavancin has a comparable efficacy as vancomycin since vancomycin dose was inadequate.

There was minimal data available regarding the effectiveness of novel glycopeptides for the treatment of patients with bacteremia. Only 150 CE patients were analysed in 3 trials. Telavancin was not less effective than vancomycin in two trials studying telavancin, while two doses of dalbavancin achieved a significantly higher response rate than a course of 14-days twice-daily parenteral vancomycin. This finding was supported by a recent retrospective cohort study [48]. We should address that trials included in the analysis were open-label trials. However, physicians will continue to treat patients suffering from bloodstream infections based on limited evidence on novel antibiotics efficacy, as there are limited options for the management of this life-threatening infection caused by *Staphylococcus aureus* including MRSA.

Regarding osteomyelitis, only one trial compared dalbavancin to standard of care, most commonly IV vancomycin. The dalbavancin regimen used was 1500 mg on day 1 and 1500 mg on day 8 to provide longer duration of coverage of approximately 4–6 weeks (which is different from the ABSSSI regimen of 1500 mg on day 1 or 1000 mg on day 1 and 500 mg on day 8 which provide coverage for 14 days for ABSSSI). Ninety seven percent in the CE, and mITT populations had high clinical cure rates at day 42 with a sustained effect for 1 year. An important issue observed in this trial should be addressed, as dalbavancin can significantly reduce the mean length of hospital stay (P: < 0.001). In this regard, dalbavancin is considered a convenient and effective option for an infection that has been historically challenging for patients, providers, and the health care system. Moreover, several recent retrospective studies reported similar clinical and microbiological response rates and safety profile outcomes of dalbavancin in treating patients with osteomyelitis [49–53]. Although oritavancin is not recommended for indications other than SSTIs, it has shown promise in the treatment of prosthetic joint infections caused by VRE [54, 55]. The results of a recent systematic review [56] supported the safety and efficacious effect of both dalbavancin and oritavancin for osteoarticular infection.

There are specific limitations in our meta-analysis that should be considered when interpreting the findings. First, studies that met the criterion of inclusion had different methodological quality, open-label or double blind, with appropriate or inappropriate allocation concealment. Second, ClinicalTrials.gov was searched for unpublished completed trials, and only one open-label, randomized phase III trial [24] was found, but the authors were not contacted to obtain missing data from the original report. Finally, telavancin was under-dosed in two trials [19, 24]. The PRISMA checklist is presented in S1 Checklist.

## Conclusion

Our meta-analysis demonstrates that novel glycopeptides are as effective as vancomycin in the treatment of patients with SSTIs, hospital acquired pneumonia, bacteremia and osteomyelitis. Telavancin is considered a potential alternative in the case of serious MRSA infections that are difficult to treat by standard antibiotics. However, the efficacy of telavancin should be weighed against safety during clinical use in the treatment of any infection. Furthermore, a regimen including one or two doses of dalbavancin or one dose of oritavancin constitute promising and suitable alternatives for the treatment of serious infections (that need a long period of hospitalization) in different clinical settings including outpatient settings such as emergency rooms and infusion centers. Hence, this may improve the quality care as a consequence of lowering overall expenditure. Finally, randomized trials with high methodological quality are warranted to confirm the efficacy and safety of these antibiotics in the treatment of patients with HAP, bacteremia and osteomyelitis.

## Supporting information

S1 Checklist PRISMA 2009 checklist. (DOC)

S1 File (DOCX)
