# Supplementary material for: Efficacy and safety of novel glycopeptides versus vancomycin for the treatment of gram-positive bacterial infections including methicillin resistant Staphylococcus aureus: A systematic review and meta-analysis
Source: PLoS One. 2021 Nov 29;16(11):e0260539. doi: 10.1371/journal.pone.0260539 (PMC8629313; doi:10.1371/journal.pone.0260539)
Supplement: S1 File — (DOCX) [file pone.0260539.s002.docx]

**Treatment success in modified intention to treat population at TOC**

| **study** | **event** | **total** | **event** | **Total** |
| --- | --- | --- | --- | --- |
| Stryjewski et al, 2005 | 66 | 84 | 66 | 83 |
| Stryjewski et al, 2006 | 82 | 100 | 81 | 95 |
| Stryjewski et al, 2008 | 710 | 929 | 697 | 938 |
| Rubinstein et al, 2011 | 441 | 749 | 449 | 754 |
| Stryjewski et al, 2014 | 8 | 29 | 11 | 29 |
| Rappo U et al, 2018 | 65 | 67 | 7 | 8 |
| Corey et al, 2014 | 378 | 473 | 383 | 479 |
| Corey et al, 2015 | 416 | 503 | 404 | 502 |

**Treatment success in clinical evaluable population at TOC**

| study | event | Total | event | Total |
| --- | --- | --- | --- | --- |
| Stryjewski et al, 2005 | 66 | 72 | 66 | 69 |
| Stryjewski et al, 2006 | 74 | 77 | 72 | 77 |
| Stryjewski et al, 2008 | 658 | 745 | 648 | 744 |
| Rubinstein et al, 2011 | 257 | 312 | 276 | 342 |
| Stryjewski et al, 2014 | 7 | 8 | 9 | 9 |
| NCT02208063 | 22 | 47 | 27 | 52 |
| Raad I et al, 2005 | 13 | 14 | 9 | 20 |
| Boucher et al, 2014 | 495 | 520 | 477 | 501 |
| Rappo U et al, 2018 | 65 | 67 | 7 | 8 |
| Corey et al, 2014 | 357 | 394 | 352 | 397 |
| Corey et al, 2015 | 398 | 427 | 387 | 408 |

**Treatment success in microbiological evaluable population based on the total number of staphylococcus aureus at TOC**

| **Study** | **event** | **total** | **event** | **Total** |
| --- | --- | --- | --- | --- |
| Stryjewski et al, 2005 | 52 | 56 | 53 | 56 |
| Stryjewski et al, 2006 | 62 | 64 | 53 | 57 |
| Stryjewski et al, 2008 | 484 | 539 | 490 | 563 |
| Rubinstein et al, 2011 | 192 | 243 | 182 | 237 |
| Stryjewski et al, 2014 | 7 | 8 | 8 | 9 |
| NCT02208063 | 26 | 47 | 31 | 52 |
| Boucher et al, 2014 | 176 | 191 | 166 | 177 |
| Rappo U et al, 2018 | 41 | 42 | 5 | 6 |

**Treatment success in microbiological evaluable population infected with MRSA at TOC**

| **Study** | **event** | **total** | **event** | **total** |
| --- | --- | --- | --- | --- |
| Stryjewski et al, 2006 | 25 | 26 | 17 | 19 |
| Stryjewski et al, 2008 | 252 | 278 | 260 | 301 |
| Stryjewski et al, 2014 | 5 | 5 | 4 | 4 |
| Boucher et al, 2014 | 67 | 74 | 48 | 50 |
| Rappo U et al, 2018 | 4 | 4 | 1 | 1 |

**Early clinical response at (48-72h) to oritavancin in modified intention to treat population**

| **Study** | **event** | **total** | **event** | **total** |
| --- | --- | --- | --- | --- |
| Corey et al, 2014 | 391 | 475 | 378 | 479 |
| Corey et al, 2015 | 403 | 503 | 416 | 502 |

**Early clinical response at (48-72h) to dalbavancin in intention to treat population**

| **study** | **event** | **total** | **event** | **total** |
| --- | --- | --- | --- | --- |
| Boucher et al, 2014 | 525 | 659 | 521 | 653 |

**Microbiological success in microbiological evaluable population infected with MRSA at TOC**

| **Study** | **event** | **total** | **event** | **total** |
| --- | --- | --- | --- | --- |
| Stryjewski et al, 2005 | 16 | 19 | 14 | 19 |
| Stryjewski et al, 2006 | 24 | 26 | 13 | 19 |
| Stryjewski et al, 2008 | 250 | 278 | 257 | 301 |
| Rubinstein et al, 2011 | 104 | 139 | 115 | 154 |
| Stryjewski et al, 2014 | 5 | 5 | 3 | 4 |

**Microbiological success in microbiological evaluable population infected with MSSA at TOC**

| **study** | **event** | **total** | **event** | **Total** |
| --- | --- | --- | --- | --- |
| Stryjewski et al, 2008 | 161 | 181 | 157 | 176 |
| Rubinstein et al, 2011 | 51 | 58 | 27 | 36 |
| Stryjewski et al, 2014 | 2 | 3 | 4 | 5 |

**Adverse events in modified intention to treat population**

| **study** | **event** | **total** | **Event** | **Total** |
| --- | --- | --- | --- | --- |
| Stryjewski et al, 2005 | 47 | 84 | 50 | 83 |
| Stryjewski et al, 2006 | 56 | 100 | 54 | 95 |
| Stryjewski et al, 2008 | 735 | 929 | 676 | 938 |
| Rubinstein et al, 2011 | 616 | 751 | 613 | 752 |
| Stryjewski et al, 2014 | 26 | 29 | 21 | 29 |
| NCT02208063 | 29 | 58 | 25 | 60 |
| Raad I et al, 2005 | 33 | 33 | 31 | 34 |
| Boucher et al, 2014 | 139 | 652 | 183 | 651 |
| Rappo U et al, 2018 | 10 | 70 | 0 | 10 |
| Corey et al, 2014 | 108 | 473 | 151 | 481 |
| Corey et al, 2015 | 109 | 503 | 128 | 502 |

**Serious adverse events in modified intention to treat population**

| **study** | **event** | **total** | **event** | **Total** |
| --- | --- | --- | --- | --- |
| Stryjewski et al, 2005 | 4 | 84 | 9 | 83 |
| Stryjewski et al, 2006 | 7 | 100 | 3 | 95 |
| Stryjewski et al, 2008 | 69 | 929 | 42 | 938 |
| Rubinstein et al, 2011 | 234 | 751 | 197 | 752 |
| Stryjewski et al, 2014 | 11 | 29 | 6 | 29 |
| NCT02208063 | 16 | 58 | 13 | 60 |
| Raad I et al, 2005 | 0 | 33 | 1 | 34 |
| Boucher et al, 2014 | 2 | 652 | 4 | 651 |
| Rappo U et al, 2018 | 2 | 70 | 0 | 10 |
| Corey et al, 2014 | 3 | 473 | 3 | 481 |
| Corey et al, 2015 | 22 | 503 | 23 | 502 |

**Discontinuation due to adverse events in modified intention to treat population**

| **study** | **event** | **total** | **event** | **Total** |
| --- | --- | --- | --- | --- |
| Stryjewski et al, 2005 | 5 | 84 | 4 | 83 |
| Stryjewski et al, 2006 | 6 | 100 | 3 | 95 |
| Stryjewski et al, 2008 | 73 | 929 | 53 | 938 |
| Rubinstein et al, 2011 | 60 | 751 | 40 | 752 |
| Stryjewski et al, 2014 | 2 | 29 | 2 | 29 |
| Raad I et al, 2005 | 0 | 33 | 5 | 34 |
| Boucher et al, 2014 | 14 | 652 | 13 | 651 |
| Rappo U et al, 2018 | 0 | 70 | 0 | 10 |
| Corey et al, 2014 | 29 | 473 | 41 | 481 |
| Corey et al, 2015 | 18 | 503 | 13 | 502 |

**Mortality rate in modified intention to treat population**

| **Study** | **event** | **total** | **event** | **total** |
| --- | --- | --- | --- | --- |
| Stryjewski et al, 2006 | 0 | 100 | 1 | 95 |
| Stryjewski et al, 2008 | 8 | 929 | 8 | 938 |
| Rubinstein et al, 2011 | 150 | 751 | 140 | 752 |
| Stryjewski et al, 2014 | 5 | 29 | 3 | 29 |
| NCT02208063 | 3 | 58 | 5 | 60 |
| Boucher et al, 2014 | 1 | 652 | 7 | 651 |
| Rappo U et al, 2018 | 1 | 70 | 0 | 10 |
| Corey et al, 2014 | 1 | 473 | 2 | 481 |
| Corey et al, 2015 | 1 | 503 | 1 | 502 |

**Laboratory abnormalities**

**Liver function test**

| **Study** | **event** | **total** | **event** | **total** |
| --- | --- | --- | --- | --- |
| Stryjewski et al, 2005 | 13 | 84 | 15 | 83 |
| Stryjewski et al, 2006 | 10 | 100 | 31 | 95 |
| Stryjewski et al, 2008 | 29 | 929 | 50 | 938 |
| Rubinstein et al, 2011 | 45 | 751 | 50 | 752 |
| Stryjewski et al, 2014 | 4 | 29 | 1 | 29 |
| NCT02208063 | 1 | 58 | 1 | 60 |
| Corey et al, 2014 | 11 | 473 | 5 | 481 |
| Corey et al, 2015 | 27 | 503 | 21 | 502 |

**Serum creatinine**

| Study | event | Total | event | total |
| --- | --- | --- | --- | --- |
| Stryjewski et al, 2005 | 7 | 84 | 2 | 83 |
| Stryjewski et al, 2006 | 5 | 100 | 0 | 95 |
| Stryjewski et al, 2008 | 52 | 929 | 19 | 938 |
| Rubinstein et al, 2011 | 111 | 751 | 69 | 752 |
| Stryjewski et al, 2014 | 5 | 29 | 2 | 29 |
| NCT02208063 | 1 | 58 | 0 | 60 |

**Hypokalemia**

| **Study** | **event** | **total** | **event** | **total** |
| --- | --- | --- | --- | --- |
| Stryjewski et al, 2006 | 7 | 100 | 0 | 95 |
| Stryjewski et al, 2008 | 16 | 929 | 7 | 938 |
| Rubinstein et al, 2011 | 50 | 751 | 37 | 752 |
| Stryjewski et al, 2014 | 3 | 29 | 1 | 29 |
| NCT02208063 | 10 | 58 | 8 | 60 |
| Raad I et al, 2005 | 6 | 33 | 0 | 34 |

**Anemia**

| **Study** | **Event** | **total** | **event** | **total** |
| --- | --- | --- | --- | --- |
| Stryjewski et al, 2005 | 8 | 84 | 8 | 83 |
| Stryjewski et al, 2006 | 13 | 100 | 14 | 95 |
| Rubinstein et al, 2011 | 64 | 751 | 85 | 752 |
| Stryjewski et al, 2014 | 3 | 29 | 2 | 29 |
| NCT02208063 | 6 | 58 | 7 | 60 |
| Raad I et al, 2005 | 6 | 33 | 4 | 34 |

**Leukopenia**

| **Study** | **event** | **total** | **event** | **total** |
| --- | --- | --- | --- | --- |
| Stryjewski et al, 2005 | 1 | 84 | 2 | 83 |
| Stryjewski et al, 2006 | 2 | 100 | 1 | 95 |
| Stryjewski et al, 2008 | 8 | 929 | 10 | 938 |
| Rubinstein et al, 2011 | 1 | 751 | 6 | 752 |

**Detailed adverse events**

**Nausea**

| **Study** | **event** | **total** | **event** | **total** |
| --- | --- | --- | --- | --- |
| Stryjewski et al, 2005 | 13 | 84 | 11 | 83 |
| Stryjewski et al, 2006 | 16 | 100 | 6 | 95 |
| Stryjewski et al, 2008 | 249 | 929 | 142 | 938 |
| Rubinstein et al, 2011 | 40 | 751 | 31 | 752 |
| Stryjewski et al, 2014 | 1 | 29 | 3 | 29 |
| NCT02208063 | 6 | 58 | 6 | 60 |
| Raad I et al, 2005 | 3 | 33 | 4 | 34 |
| Boucher et al, 2014 | 16 | 652 | 19 | 651 |
| Corey et al, 2014 | 52 | 473 | 43 | 481 |
| Corey et al, 2015 | 45 | 503 | 60 | 502 |

**Vomiting**

| **Study** | **event** | **total** | **event** | **total** |
| --- | --- | --- | --- | --- |
| Stryjewski et al, 2005 | 8 | 84 | 3 | 83 |
| Stryjewski et al, 2006 | 8 | 100 | 6 | 95 |
| Stryjewski et al, 2008 | 127 | 929 | 69 | 938 |
| Stryjewski et al, 2014 | 1 | 29 | 3 | 29 |
| Raad I et al, 2005 | 2 | 33 | 7 | 34 |
| Corey et al, 2014 | 23 | 473 | 18 | 481 |
| Corey et al, 2015 | 22 | 503 | 28 | 502 |

**Constipation**

| **Study** | **event** | **total** | **event** | **total** |
| --- | --- | --- | --- | --- |
| Stryjewski et al, 2005 | 3 | 84 | 5 | 83 |
| Stryjewski et al, 2006 | 5 | 100 | 7 | 95 |
| Stryjewski et al, 2008 | 96 | 929 | 61 | 938 |
| Rubinstein et al, 2011 | 70 | 751 | 71 | 752 |
| NCT02208063 | 3 | 58 | 7 | 60 |
| Raad I et al, 2005 | 6 | 33 | 4 | 34 |
| Corey et al, 2014 | 19 | 473 | 21 | 481 |
| Corey et al, 2015 | 14 | 503 | 17 | 502 |

**Diarrhea**

| **Study** | **event** | **total** | **event** | **total** |
| --- | --- | --- | --- | --- |
| Stryjewski et al, 2006 | 6 | 100 | 5 | 95 |
| Stryjewski et al, 2008 | 67 | 929 | 76 | 938 |
| Rubinstein et al, 2011 | 85 | 751 | 92 | 752 |
| Stryjewski et al, 2014 | 0 | 29 | 2 | 29 |
| NCT02208063 | 3 | 58 | 6 | 60 |
| Raad I et al, 2005 | 7 | 33 | 4 | 34 |
| Boucher et al, 2014 | 5 | 652 | 16 | 651 |
| Corey et al, 2014 | 23 | 473 | 17 | 481 |
| Corey et al, 2015 | 13 | 503 | 15 | 502 |

**Headache**

| **Study** | **event** | **total** | **event** | **total** |
| --- | --- | --- | --- | --- |
| Stryjewski et al, 2005 | 9 | 84 | 8 | 83 |
| Stryjewski et al, 2006 | 8 | 100 | 4 | 95 |
| Stryjewski et al, 2008 | 130 | 929 | 120 | 938 |
| Stryjewski et al, 2014 | 3 | 29 | 3 | 29 |
| NCT02208063 | 3 | 58 | 3 | 60 |
| Corey et al, 2014 | 34 | 473 | 38 | 481 |
| Corey et al, 2015 | 35 | 503 | 28 | 502 |

**Pruritus**

| Study | event | total | event | total |
| --- | --- | --- | --- | --- |
| Stryjewski et al, 2006 | 6 | 100 | 8 | 95 |
| Stryjewski et al, 2008 | 35 | 929 | 43 | 938 |
| Stryjewski et al, 2014 | 2 | 29 | 3 | 29 |
| Boucher et al, 2014 | 4 | 652 | 15 | 651 |
| Corey et al, 2014 | 16 | 473 | 44 | 481 |
| Corey et al, 2015 | 13 | 503 | 29 | 502 |
